# Heat Transfer and Friction Characteristics of the Microfluidic Heat Sink with Variously-Shaped Ribs for Chip Cooling

**DOI:** 10.3390/s150409547

**Published:** 2015-04-22

**Authors:** Gui-Lian Wang, Da-Wei Yang, Yan Wang, Di Niu, Xiao-Lin Zhao, Gui-Fu Ding

**Affiliations:** Key Laboratory of Science and Technology on Micro/Nano Fabrication, Shanghai Jiao Tong University, No. 800 Dong Chuan Road, Shanghai 200240, China; E-Mails: wglshk@sjtu.edu.cn (G.-L.W.); davidyoung2@sjtu.edu.cn (D.-W.Y.); wyyw@sjtu.edu.cn (Y.W.); niudi1133409031@sjtu.edu.cn (D.N.); xlzhao@sjtu.edu.cn (X.-L.Z.)

**Keywords:** microfluidic heat sink, micro-rib, heat transfer, friction factor

## Abstract

This paper experimentally and numerically investigated the heat transfer and friction characteristics of microfluidic heat sinks with variously-shaped micro-ribs, *i.e.*, rectangular, triangular and semicircular ribs. The micro-ribs were fabricated on the sidewalls of microfluidic channels by a surface-micromachining micro-electro-mechanical system (MEMS) process and used as turbulators to improve the heat transfer rate of the microfluidic heat sink. The results indicate that the utilizing of micro-ribs provides a better heat transfer rate, but also increases the pressure drop penalty for microchannels. Furthermore, the heat transfer and friction characteristics of the microchannels are strongly affected by the rib shape. In comparison, the triangular ribbed microchannel possesses the highest Nusselt number and friction factor among the three rib types.

## 1. Introduction

Due to extensive growth in the power density of electronic chips and miniaturization of electronic packages, the microfluidic heat sinks based on micro-electro-mechanical system (MEMS) technology are drawing more and more attention as an advanced cooling approach. The MEMS microfluidic heat sinks can be directly packaged with the electronic chips and dissipate a large amount of heat flux within a relatively small area. The concept of microfluidic cooling technology was introduced firstly by Tuckerman and Pease [1]. They demonstrated that a heat flux as high as 790 W/cm^2^ can be removed with a maximum substrate temperature rise of 71 °C.

In order to further enhance the cooling capacity of microfluidic heat sinks, nanoparticles with high thermal conductivity have been proposed to be added in the coolant fluid. Yi *et al.* demonstrated that Al_2_O_3_ [2], CrO_2_ [3] and Fe_2_O_3_ [4] nanoparticles all could enhance the overall thermal conductivity of the coolant fluid. In addition, setting up periodic rib turbulators on the microchannel walls is also a widely-used technique for improving the heat transfer performance of microfluidic heat sinks. The ribs can disturb boundary layers and promote recirculation flow to enhance the heat convection effect between the channel wall and cooling water flow. In the past couple of decades, the cooling performance of ribbed microchannels has been studied by a large number of researchers. Wee *et al.* numerically studied the Nusselt number distribution of heat sinks with smooth, ribbed and grooved surfaces. They found that the heat sinks with rib turbulators possessed the highest Nusselt number [5]. Desrues *et al.* also carried out a numerical investigation on heat transfer in three-dimensional channels with ribs. They agreed that the microchannel with ribs had a better heat transfer performance compared to a convectional smooth microchannel [6]. Cheng numerically investigated the two-layer microchannel heat sink with ribs. Compared with a single- and two-layer smooth microchannel, the two-layer microchannel with ribs had a lower thermal resistance [7].

However, the presence of ribs in the microchannels not only increases the heat transfer rate, but also induces a greater pressure loss. Therefore, some investigations have been carried out to obtain optimized arrangement and dimension parameters, which can balance the pressure loss and heat transfer for a microfluidic heat sink. Chai *et al.* performed numerical research about an interrupted microchannel heat sink with rectangular ribs in transverse microchambers. Their findings indicated that the new interrupted microchannel with a rib length of 0.5 mm provided the highest enhancement factor. At the same time, there was an optimum operation scope for the new interrupted microchannel with a fixed rib width value [8].

During the past few decades, the rib shape in the channel is attracting more and more attention. Ahn *et al.* used the large eddy simulation technique to investigate the effect of square and semicircle ribs on flow and heat transfer in a channel. The results showed that two ribs produced nearly the same heat transfer, but the semicircle rib yielded a lower pressure drop than the square one [9]. Kamali *et al.* performed numerical simulations to analyze the turbulent heat transfer and friction characteristics in a square duct with variously-shaped ribs mounted on one wall. The results showed that features of the inter-rib distribution of the heat transfer coefficient were strongly affected by the rib shape, and trapezoidal ribs with decreasing height in the flow direction provided the highest heat transfer enhancement and pressure drop [10]. Zhang *et al.* conducted a numerical simulation to study the laminar flow and heat transfer of the microchannels with semicircular, triangular and rectangular ribs. They found that the effects of triangular roughness elements on laminar flow and heat transfer were similar to those caused by semicircular roughness elements and much stronger than that of the rectangular roughness element [11].

From the literature review above, many numerical studies have investigated the heat transfer performance of microchannels with different ribs, but few experiments have been carried out to further verify the heat transfer and friction performance of ribbed microchannels. In addition, alternated opposed ribs with various shapes are seldom proposed in the microchannels. Thus, the main aim of the present work is to experimentally and numerically study the heat transfer and friction characteristics of the microchannels with different alternated opposed ribs. In this paper, a series of experiments and simulations are performed for different ribbed microchannels to study and compare their heat transfer and friction characteristics. Meanwhile, the mechanisms of flow and heat transfer in ribbed microchannels are fully revealed in this paper.

## 2. Problem Description

**Figure 1:**
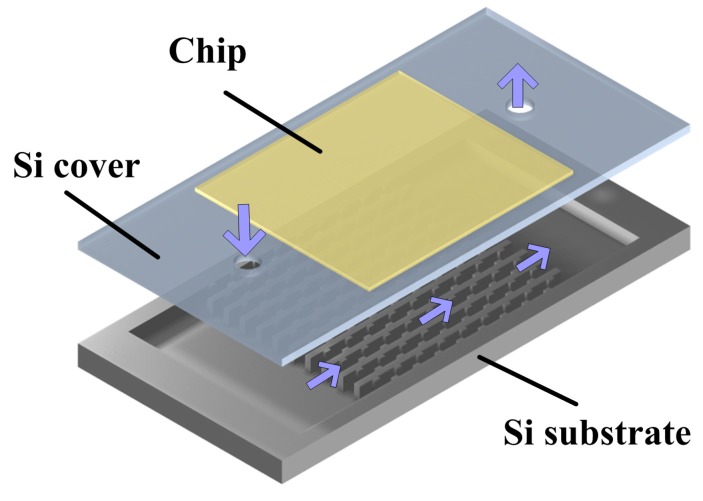
Schematic of the microfluidic heat sink.

**Figure 2:**
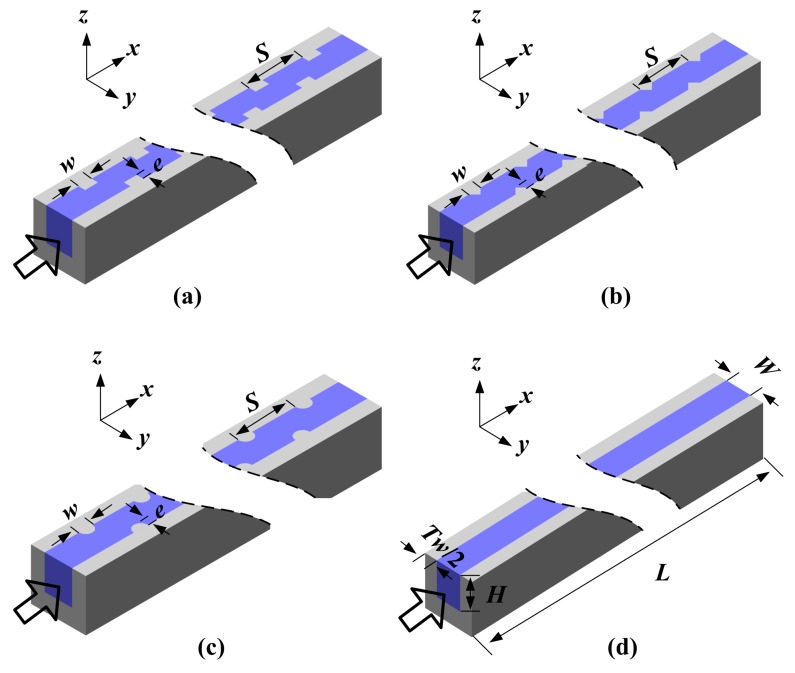
(**a**) The rectangular ribbed microchannel; (**b**) the triangular ribbed microchannel; (**c**) the semicircular ribbed microchannel; and (**d**) the conventional smooth microchannel.

The microfluidic heat sink presented in this paper consists of two parts, namely a silicon (Si) cover and a Si substrate with thirteen parallel microchannels, as shown in Figure 1. The electronic chip can be directly mounted on the top of the Si cover. The total length, width and height of the heat sink are 20 mm, 12 mm and 1.45 mm, respectively. Additionally, the single microchannel has a length of 10 mm (*L*), a width of 0.425 mm (*W*) and a height of 0.5 mm (*H*). The wall surface of the microchannels is roughened with variously-shaped ribs, namely rectangular, triangular and semicircular ribs. Figure 2 shows single-branch ribbed microchannels and a conventional smooth microchannel, respectively. As shown in Figure 2, these ribs are arranged on the right and left sidewalls in a staggered configuration and have the same rib height (*e*), width (*w*) and spacing (*S*). The geometrical parameter details of the microchannels in the present study are listed in Table 1.

**Table 1:** Geometry details of the microchannels.

Characteristic	Values (μm)
Channel width *W*	425
Channel height *H*	500
Channel length *L*	10,000
Thickness of the wall *t_w_*	300
Cover height *h_c_*	450
Substrate height *h_s_*	1000
Rib height *e*	100
Rib width *w*	200
Rib spacing *S*	800

## 3. Experimental Work

### 3.1. Fabrication

**Figure 3:**
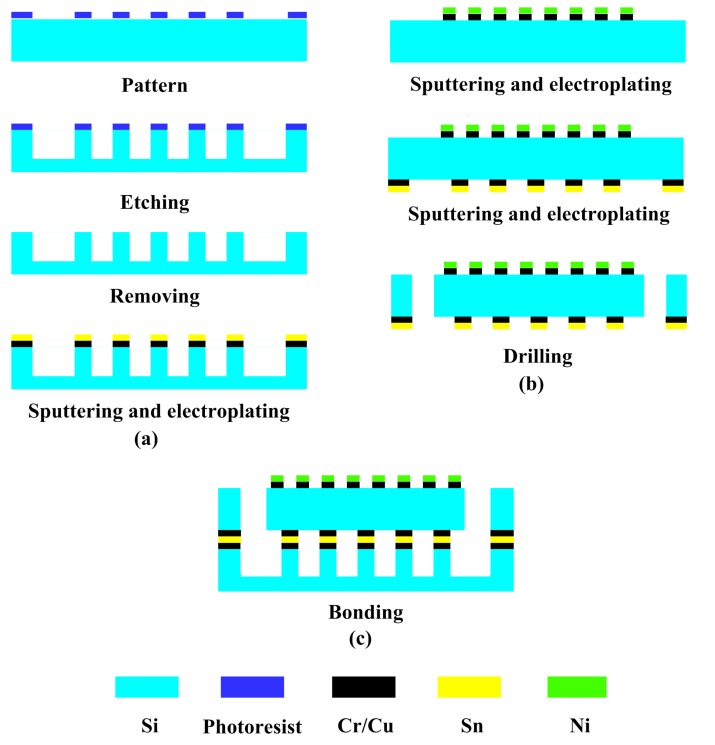
Main fabrication process of the microfluidic heat sink with variously-shaped ribs.

**Figure 4:**
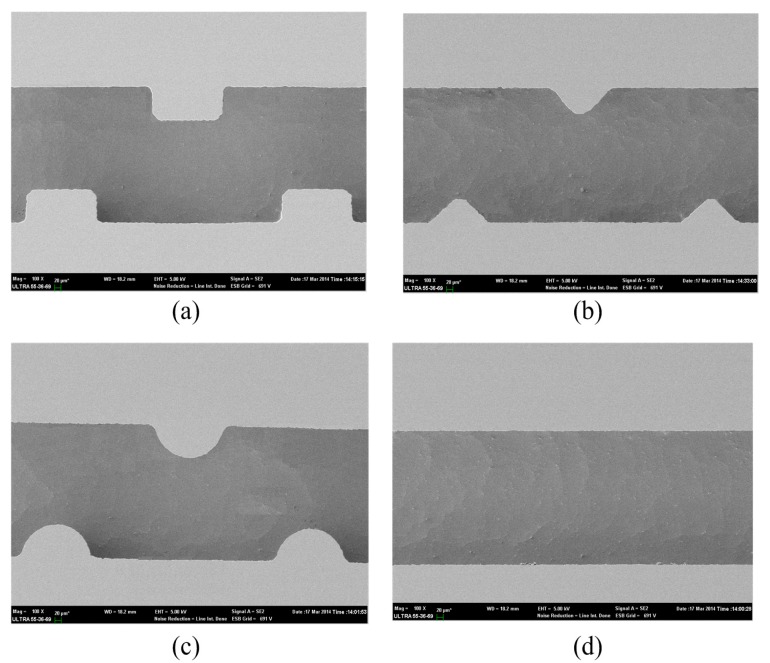
The SEM of (**a**) the rectangular ribbed microchannel, (**b**) the triangular ribbed microchannel, (**c**) the semicircular ribbed microchannel and (**d**) the smooth microchannel.

The main fabrication process of microfluidic heat sinks with different ribs is shown in Figure 3. As shown in Figure 3a, an N-type Si wafer with a thickness of 1 mm was used as the starting substrate for manufacturing the Si substrate. Firstly, a layer of photoresist was spin coated on the front side and then patterned by a UV lithographic process. Subsequently, the inductively-coupled plasma-reactive ion etching (ICP-RIE) process was performed 3 h to etch the exposed Si. These processes mentioned above created ribbed microchannels with a depth of 500 µm. The scanning electron micrograph (SEM) of the ribbed and smooth microchannels is shown in Figure 4. After removing the photoresist by acetone solution, a 100 Å-thick layer of chromium (Cr) was deposited on the front side of the wafer to enhance the adhesion characteristic and was followed by sputtering a 600 Å-thick layer of copper (Cu). Finally, a 20-µm thickness of tin (Sn) film serving as the bonding layer was created through UV lithography and electroplating processes.

Another Si wafer with a thickness of 450 µm was processed to fabricate the Si cover, as shown in Figure 3b. A Cr/Cu seed layer was sputtered on the front side of the wafer. Then, a nickel (Ni) thin-film heater was created by the electroplating process as a heat source. In addition, another Sn layer was also formed on the back side of the wafer. Eventually, the inlet and outlet of the microfluidic heat sinks were fabricated by an excimer laser.

**Figure 5:**
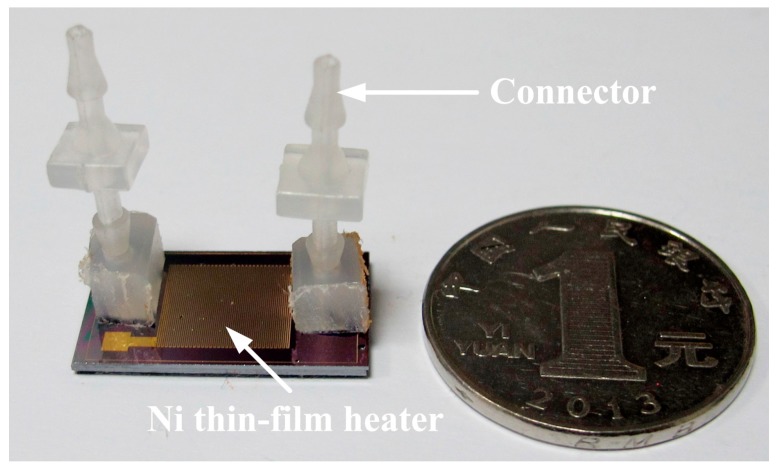
The prototype of the test device.

The prepared cover and substrate were then eutectically bonded together to form a sealed device, as shown in Figure 3c. The precise assembly process of the cover and substrate was performed using a Micro Placer (OK Industries MP-2000 Series@, Chicago, IL, USA). Afterwards, the inflow/outflow pipe connectors were fixed on the inlet/outlet ports through the PDMS fixed support. A photograph of the test device is shown in Figure 5.

### 3.2. Experimental Setup

**Figure 6:**
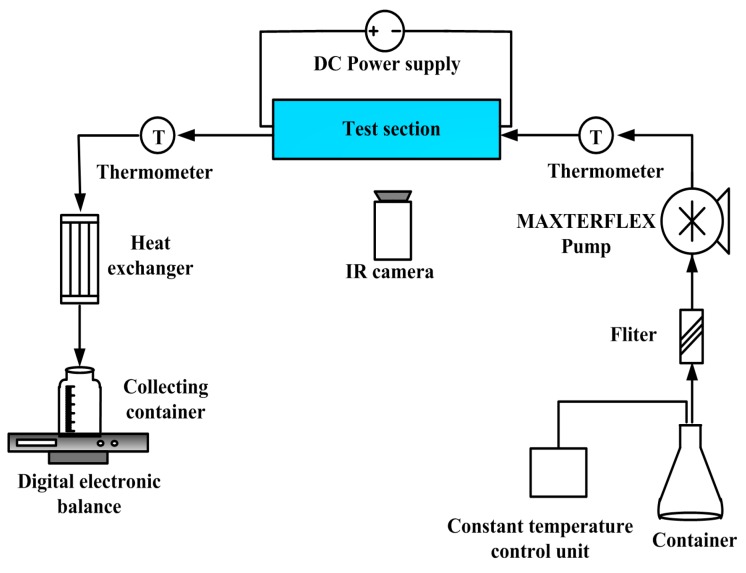
Schematic of the experimental setup.

Figure 6 shows an experimental setup and the corresponding apparatus. The de-ionized water (DI water) as the working fluid was fed from a container and then purified by a filter to remove the contaminants. The temperature of DI water in the container was controlled by a constant temperature control unit with an uncertainty of ±1 °C. The flow rate was controlled by a MASTERFLEX tubing pump and affirmed by weighing the mass increment over a longer given period of time using a high precision electronic balance (made by Sartorius, Model TE214S, accuracy: 0.002 g, Goettingen, Germany). The measurements of temperature and pressure drop were carried out at the inlet and outlet by two thermometers (made by Omega, Model DP-251, accuracy: 0.01 °C) and piezometers (made by Omega, Model DP-251, accuracy: 0.05%), respectively.

By a DC power supply, the Ni thin-film heater was driven to provide a uniform heat flux. The generated heat was then transferred to the working fluid flowing through the microchannels. A high accuracy infrared radiator imaging system (FLIR Thermal CAM PM595 IR, Boston, MA, USA) was used to measure the temperature of the top surface of the microfluidic heat sink. To obtain a good test accuracy, a very thin “black lacquer” with an emissivity of approximately 0.93 was uniformly spurted onto the test surface.

## 4. Numerical Work

### 4.1. Computational Domain

To fully understand the liquid flow and heat transfer in the ribbed microchannels, a numerical 3D conjugate heat transfer simulation was simultaneously carried out with the finite-volume-based computational fluid dynamics (CFD) software package, FLUENT. Due to the symmetric and periodic arrangement, single-branch microchannels were chosen as the computational domain to reduce the grid number and computational time, as shown in Figure 2. In this picture, the hollow arrows show the flow direction of cooling water, and the coordinates *x*, *y*, *z* represent streamwise, spanwise and normal direction, respectively.

### 4.2. Governing Equations and Boundary Conditions

The following assumptions were made to simplify the analysis:

(1) The fluid flow is steady and incompressible, and laminar flow prevails across the microchannels.
(2) The solid and fluid properties are constant.
(3) The effect of gravity, radiation heat transfer and viscous dissipation are negligible.

Based on the above assumptions, non-dimensional governing equations for the liquid region include continuity, momentum and energy equations. They can be expressed as follows [12]:

Continuity equation:

(1) ∇⋅(ρu)=0

Momentum equation:

(2) $$(u\cdot \nabla )\rho u=-\nabla p+\mu \nabla^{2}u$$

Energy equation:

(3) $$u\cdot \nabla T=\frac{\lambda }{\rho C_{p}}\nabla^{2}T$$

Energy equation in the solid domain is given by:

(4) $$\lambda \nabla^{2}T=0$$

where *ρ* is water density, *u* is the velocity at the inlet of the microchannel, *p* is pressure, *μ* is the dynamic viscosity, *λ* is the thermal conductivity of water and *C_p_* is the specific heat of the water.

Apart from the governing equations, related boundary conditions should be provided. The uniform velocity with different values and constant temperature (*T_in_* = 293 K) is applied in the inlet of the microchannel. At the exit, a pressure outlet boundary condition is specified with a fixed pressure of 1.013 × 10^5^ Pa. A uniform heat flux of 50 W/cm^2^ is applied on the top surface of the microchannel. Both lateral surfaces of the numerical domain are set as “symmetry”.

The aforesaid mass, momentum and energy governing equations are discretized using the second-order upwind scheme. The SIMPLE algorithm is employed for pressure-velocity coupling to achieve the stability of solution convergence. The governing equations are solved iteratively until they are convergent, and the convergence criterion is that the normalized residuals are less than 10^−6^ for the flow equations and 10^−8^ for the energy equation.

### 4.3. Grid Independence Test

In order to investigate the effect of grid density on the computational results, four different grid systems are studied. Their grid numbers are 154,620, 232,218, 347,421 and 425,814, respectively. The average Nusselt number and friction factor of the fourth grid system differ from those of the third by less than 0.5% and 0.6%, respectively. Hence, the third grid system with a grid number of 347,421 was finally chosen in the simulation.

## 5. Data Reduction

In this research, the Reynolds number is defined by [12]:

(5) *Re* = *ρūD_h_/μ*

where *ū* is the average velocity of the inlet, *D_h_* is the hydraulic diameter and defined by [12]:

(6) *D_h_* = 2*WH*/(*W + H*)

The thermal performance of microchannels is evaluated in terms of the Nusselt number. It is defined as [13]:

(7) $$Nu=\frac{hD_{h}}{\lambda }$$

where *h* is the heat transfer coefficient and described as [13]:

(8) $$h=\frac{Q}{NA_{ch}\Delta T}=\frac{qA_{h\text{eat}}}{2N(W+H)L\Delta T}$$

where *Q* is the total heat transfer, *q* is the heat flux, *A_heat_* is effective heating area and *A_ch_* is the contact surface area between the water and Si for a single microchannel, respectively. The mean temperature difference between the microchannel walls and the working fluid, Δ*T*, can be computed by Δ*T* = *T_w_* − (*T_in_* + *T_out_*)/2, where *T_w_*, *T_in_* and *T_out_* are the mean temperature at the top surface of the Si cover, inlet and outlet temperature, respectively. The total heat gained by the DI water can be determined from the energy balance equation below:

(9) *Q* = *ṁC_p_*(*T_out_*− *T_in_*)

Meanwhile, a layer of epoxy resin (λ = 0.2 W/m/K) was coated on the inner and outer walls of inlet and outlet manifold in order to prevent the heat loss at the inlet and outlet manifold.

The Fanning friction factor is used to evaluate the average friction factor of DI water through the microchannels, which is determined from the pressure drop measurements. The expression is given as [13]:

(10) *f* = Δ*pD_h_*/2*ρLū^2^*

In the experiments, the measured pressure drop of the test device includes the pressure drop across thirteen microchannels and the minor losses due to abrupt contraction and expansion at the inlet and outlet. Bucci *et al.* [14] have reported an experimental method to determine the pressure drop across microchannels. According to their method, another microfluidic heat sink with a longer microchannel length of 20 mm and the same inlet and outlet manifolds was manufactured. The pressure drop of two microfluidic heat sinks with a microchannel length of 20 mm and 10 mm was simultaneously tested under the same mass flow rate. Their difference was the pressure drop of 10 mm-long microchannels without pressure losses at the inlet and outlet.

The thermal enhancement factor (*η*), defined as the ratio of heat transfer coefficient of the ribbed microchannel (*h*) to that of the smooth microchannel (*h*_0_) at an equal pumping power, is widely adopted as a measure of the amount of the heat transfer enhancement against the pressure drop increase. Additionally, it is given by [15]:

(11) *η = h*/*h*_o_|*_P_* = (*Nu*/Nu_0_)/(*f*/*f_0_*)^1/3^

where *P* is the pumping power and defined by [16]:

(12) *P*=*V*∆*p*

An uncertainty analysis was carried out to give some quantitative description of the validity of test data. According to the standard error analysis [17], the uncertainties in the Reynolds number, friction factor and Nusselt number were 2.34%, 8.2% and 9.7%, respectively.

## 6. Results and Discussion

The experimental and numerical investigations were performed at *q* = 50 W/cm^2^ and *Re* = 100~1000. According to the results, the effect of rib turbulators on the flow, heat transfer and pressure drop of microchannels is analyzed as follows.

### 6.1. Flow Characteristic

**Figure 7:**
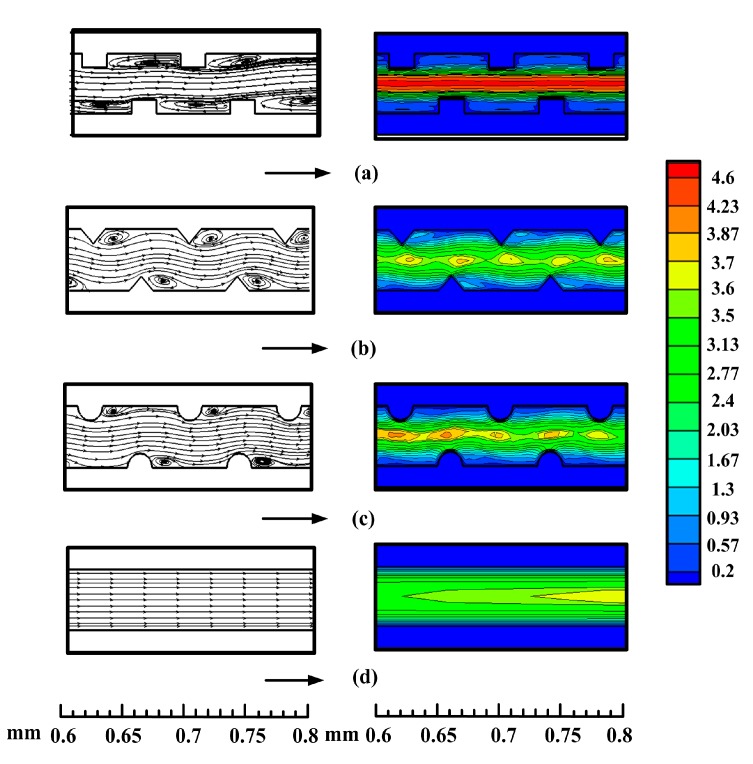
Streamline (left) and flow velocity (right) distribution along the central plane (*z* = 0.75 mm) for the (**a**) rectangular ribbed microchannel, (**b**) triangular ribbed microchannel, (**c**) semicircular ribbed microchannel and (**d**) smooth microchannel at *Re* = 1000.

Figure 7 presents the streamline (left) and flow velocity (right) distribution in the microfluidic heat sinks for *Re* = 1000 at the central plane of *z* = 0.75 mm. The black arrows show the flow direction. As shown in Figure 7, the presence of micro-ribs induces recirculation flow in the microchannels. When fluid passes over the rib, a part of the fluid impinges on the rib and then is pressed together into the microchannel with contraction of the cross-section. Due to the abrupt contraction of the flow area, the flow velocity quickly accelerates. As the fluid leaves the rib zone, the flow velocity dramatically decreases, and a transverse velocity component is produced simultaneously. The transverse velocity can transport the central core flow with lower temperature to the near-wall region. Subsequently, the recirculation is generated behind the ribs, which facilitates the mixing of the colder fluid at the center of the channel with the hotter fluid near the microchannel wall. Comparing the recirculation extent induced by three type ribs, *i.e.*, rectangular rib, triangular rib and semicircular rib, the recirculation zone induced by the triangular rib is smaller than that by rectangular rib, but slightly larger than the semicircular rib. Such a phenomenon is in agreement with a previous study reported by Zhang *et al.* [11].

### 6.2. Heat Transfer Characteristic

**Figure 8:**
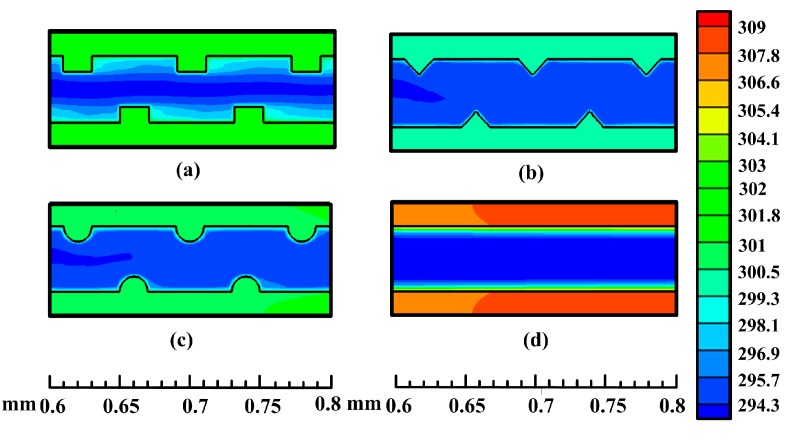
The temperature filed plots along the central plane (*z* = 0.75 mm) for the (**a**) rectangular ribbed microchannel, (**b**) triangular ribbed microchannel, (**c**) semicircular ribbed microchannel and (**d**) smooth microchannel at *Re* = 1000.

**Figure 9:**
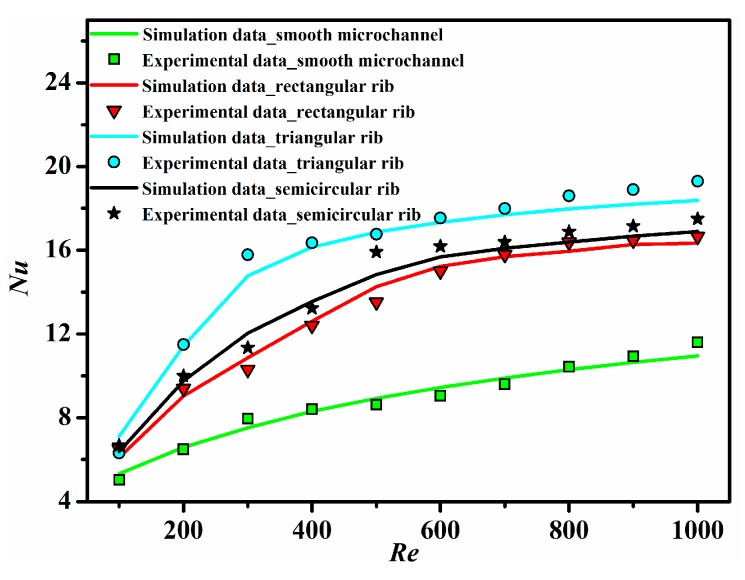
The Nusselt number as a function of the Reynolds number for all ribbed and smooth microchannels.

Figure 8 displays the temperature field in the plane of *z* = 0.75 mm at *Re* = 1000 for different ribbed microchannels and a smooth microchannel. It is obvious that the ribbed microchannels all possess lower wall temperature than the smooth microchannel. This means that the ribs have a significant influence on the temperature field. At the same time, the heat transfer characteristic of different microchannels is also presented in the form of the Nusselt number, as depicted in Figure 9. It can be seen that the experimental results of the Nusselt number match well with the numerical results, and the Nusselt number for all microchannels tends to increase with the increase of the Reynolds number with a gradually decreasing positive slope. As also can be seen from Figure 9, the heat transfer is greatly enhanced when the ribs are introduced into the microchannels. This is because the rib turbulators can induce fluid mixing and interrupt the development of the boundary layer of fluid flow. It is worth noting that the Nusselt number in the triangular ribbed microchannel is the largest, followed by that in the semicircular ribbed microchannel, and the rectangular ribbed microchannel’s is the least. Compared to the conventional smooth microchannel, their maximum enhancement of the average Nusselt number has been found to be 1.99-times, 1.85-times and 1.65-times, respectively.

### 6.3. Friction Characteristic

**Figure 10:**
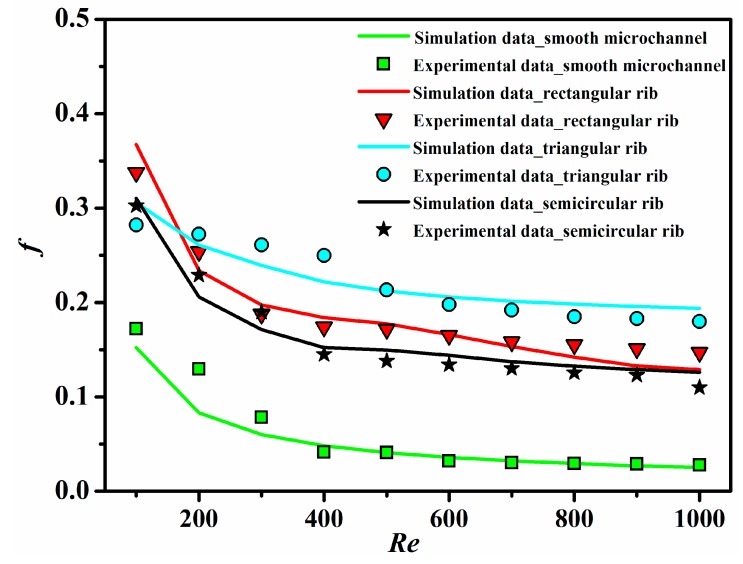
The apparent friction factor as a function of the Reynolds number for all ribbed and smooth microchannels.

Figure 10 plots the apparent friction factor as a function of the Reynolds number for all ribbed and smooth microchannels. Obviously, the apparent friction factor decreases with increasing of the Reynolds number. Meanwhile, the rib turbulators lead to a substantial increase in the apparent friction factor. In comparison with the smooth microchannel, the apparent friction factor for the rectangular ribbed microchannel, triangular ribbed microchannel and semicircular ribbed microchannel increases by 0.96~4.28-times, 0.63~6.67-times and 0.76~3.99-times, respectively. This is mainly attributed to the barrier effect of ribs for the main flow in the microchannels.

### 6.4. Thermo-Hydraulic Performance

Combining Figure 9 with Figure 10, the heat transfer enhancement in the ribbed microchannels is concerned with the penalty in terms of the increased apparent friction factor. Therefore, the performance comparison has to take into account both heat transfer and pressure drop penalties.

**Figure 11:**
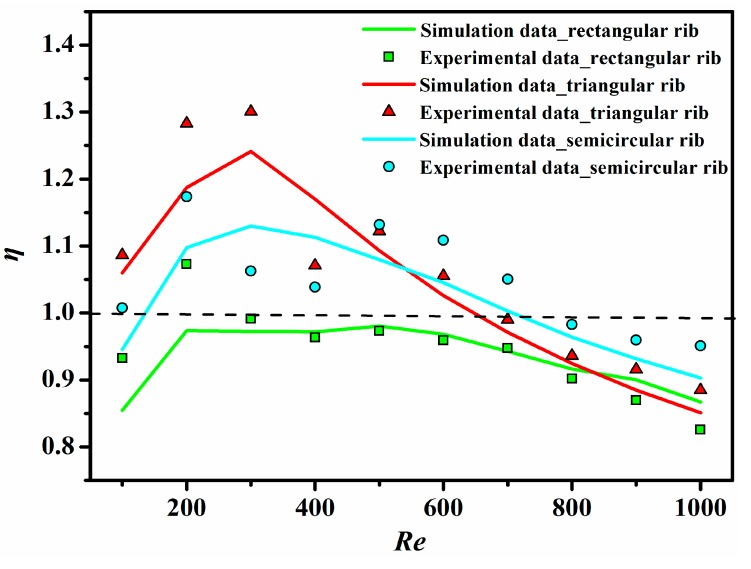
The thermal enhancement factor as a function of the Reynolds number for all ribbed microchannels.

Figure 11 shows the variation of the thermal enhancement factor (*η*) with the Reynolds number for all ribbed microchannels. It has been found that the thermal enhancement factor initially tends to increase with the rise of the Reynolds number and then decreases with the further rise of the Reynolds number for all cases. When *Re* > 600, the values for all ribbed microchannels are all lower than one, which implies that the ribbed microchannels are not superior to the conventional smooth microchannel in thermo-hydraulic performance. In addition, the triangular ribbed microchannel possesses the highest thermal enhancement factor among the three ribbed microchannels, while the rectangular ribbed microchannel has the lowest thermal enhancement factor. When 500 < *Re* < 1000, the semicircular ribbed microchannel exhibits the best thermo-hydraulic performance.

## 7. Conclusions

In this paper, heat transfer and friction characteristics for microchannels with different types of micro-ribs (including rectangular, triangular and semicircular ribs) have been numerically and experimentally investigated and compared with those of the smooth microchannel. The conclusions obtained from this study are summarized as follows:

(1) The presence of micro-ribs in the microchannels is an effective technique to improve the heat transfer characteristic.
(2) Compared to the smooth microchannel, the rectangular, triangular and semicircular ribs all provide a higher Nusselt number for microchannels, and their maximum enhancement of the average Nusselt number has been found to be 1.65-times, 1.99-times and 1.85-times, respectively.
(3) Due to the barrier effect of ribs, the apparent friction factor for rectangular ribbed microchannel, triangular ribbed microchannel and semicircular ribbed microchannel increases by 0.96~4.28-times, 0.63~6.67-times and 0.76~3.99-times, respectively.
(4) For *Re* > 600, the thermo-hydraulic performance of ribbed microchannels is not better than that of the conventional smooth microchannel. Therefore, the ribbed microchannels are suitable for the operating condition of *Re* < 600.

## Nomenclature

*A*: area, m^2^
*C_p_*: specific heat, J·kg^−1^·K^−1^
*D_h_*: hydrodynamic diameter, m
*e*: rib height, m
*f*: frictional factor
*H*: the height of a microchannel, m
*h*: heat transfer coefficient, W·m^−2^·K^−1^
*L*: the length of a microchannel, m
*ṁ*: mass flow rate, kg·s^−1^
*Nu*: Nusselt number
*P*: pumping power, W
*p*: pressure, Pa
*Q*: total heat transfer, W
*q*: heat flux, W·m^−2^
Δ*T*: temperature difference, K
*T*: temperature, K
*t*: thickness, m
*S*: pitch of the rib, m
*u*: velocity, m·s^−1^
*ū*: mean flow velocity, m·s^−1^
*V*: volumetric flow rate, m^3^·s^−1^
*W*: the width of a microchannel, m
*w*: width, m

### Greek symbols

*μ*: dynamic viscosity, Pa·s
*ρ*: density, kg·m^−3^
λ: thermal conductivity, W·m^−1^·K^−1^

### Subscripts

*c*: cover
*cr*: cross
*ch*: channel
*f*: fluid
*in*: inlet
*out*: outlet
*s*: substrate
*w*: wall
